# Editorial: Nongenomic influences of thyroid hormones and their metabolites in adults: a tribute to Mary B. Dratman

**DOI:** 10.3389/fendo.2023.1309857

**Published:** 2023-10-24

**Authors:** Pradip K. Sarkar, Joseph V. Martin

**Affiliations:** ^1^ Department of Basic Sciences, Parker University, Dallas, TX, United States; ^2^ Biology Department, Center for Computational and Integrative Biology, Rutgers University, Camden, NJ, United States

**Keywords:** thyroxine, iodothyronine, thyronamine, non-canonical, nongenomic, neurotransmitter, thyroacetic acid, thyrointegrin αvβ3 receptor

The current Research Topic is a tribute to Dr. Mary B. Dratman (see **Figure 1**), an internationally-known endocrine researcher who was instrumental in originating and promulgating the idea that many of the actions of thyroid hormones (THs) and their derivatives could be due to nongenomic effects, particularly in the brain. Dratman proposed that THs could act outside the cell nucleus, without the need to interact with the genomic apparatus, in contrast with the well-known canonical effects mediated by nuclear TH receptors.

**Figure 1:**
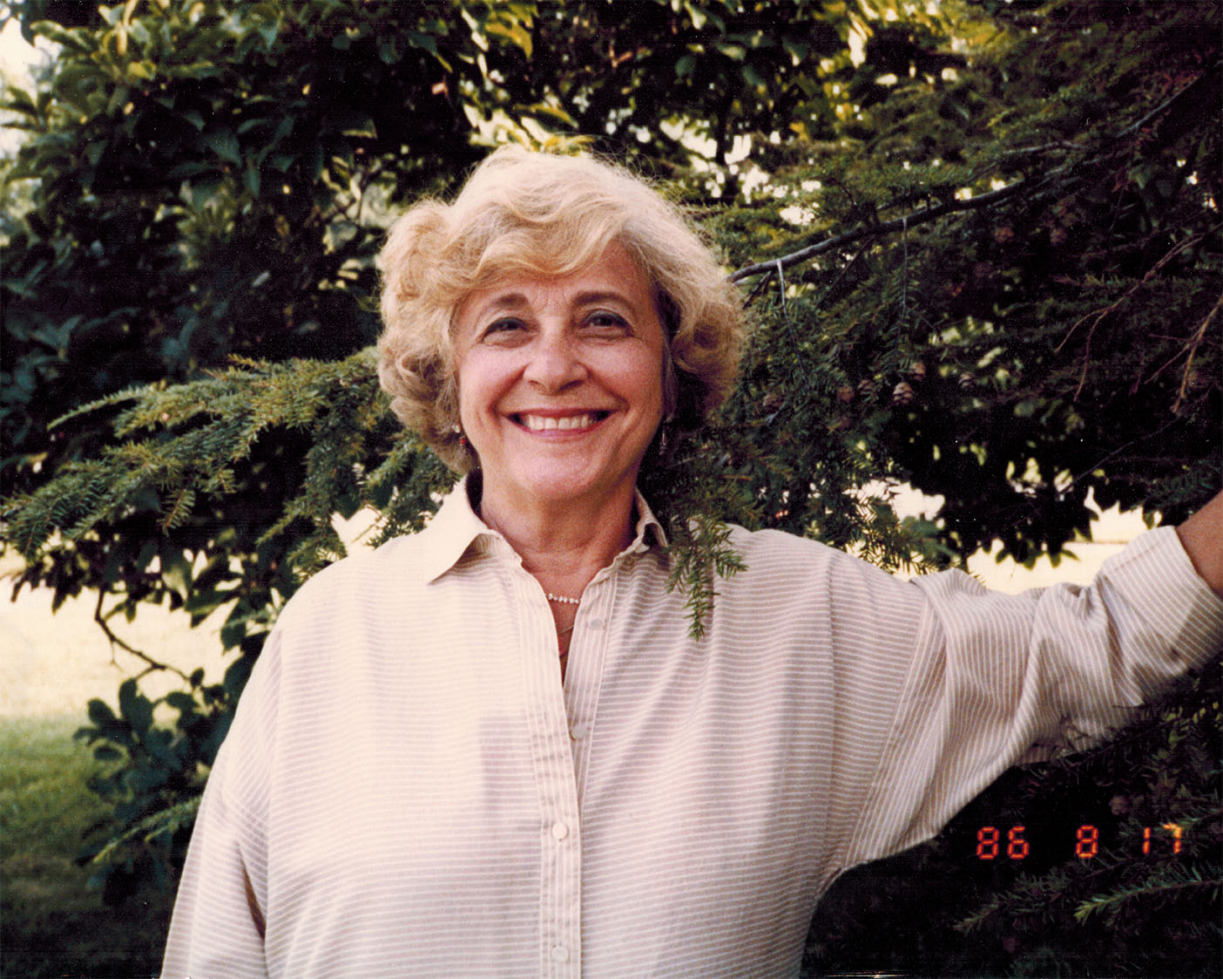
Mary Dratman in 1986. Picture reproduced with permission from Ralph Dratman, her son.

Dratman was born on July 4, 1920. She completed her undergraduate degree in chemistry from the University of Pennsylvania in 1940 and her medical degree from the Woman’s Medical College of Pennsylvania in 1945. Subsequently, she embarked on about 70 years of clinical practice specializing in thyroid endocrinology. She taught at Woman’s Medical College of Pennsylvania, the Medical College of Pennsylvania (which is now part of Drexel University College of Medicine) and at the University of Pennsylvania School of Medicine. Dratman was a beloved teacher and mentor. She inspired generations of students to pursue careers in medicine and science. She was a role model for women in medicine and a pioneer in the research field of thyroid endocrinology. She died in 2022 at the age of 101.

Dratman was an innovative and influential researcher in the field of thyroid hormone physiology of the brain, authoring over 100 papers and book chapters during her career. Chakrabarti et al. summarized the work of Dratman in relationship to the field. In 1974, Dratman published the insightful recognition that, unlike most other hormones, thyroid hormones have many actions related to their amino acid structure (1). She later made the remarkable observation that thyroid hormones are concentrated in nerve endings in the brain, indicating that these hormones might have neurotransmitter-like qualities (2). Later, a neurotransmitter- or neuromodulator-like function of THs and/or its derivative was established with the detection of 3-iodothyronamine (3-T1AM) within adrenergic systems in adult mammalian brain (3). Dratman and her coworkers published nearly half of the papers on the concepts of nongenomic actions of THs in adult mammalian brain. The illustration of unique brain-region specific accumulation of THs within the nerve terminals in adult mammals was a notable contribution by Dratman in thyroid neuroendocrinology (4).

Martin and Sarkar reviewed the literature in a different way, by characterizing the degree to which TH and its derivatives satisfy the criteria defining these compounds as neurotransmitters or neuromodulators in the adult mammalian brain. In 1996, Dratman and Gordon (5) proposed that thyroid hormone could be a neurotransmitter and in 2010, Dratman alongside her coworkers (3) proposed that 3-TAM had neurotransmitter-like properties. The criteria used by Martin and Sarkar were that the compound is present in the relevant tissue (adult mammalian brain), is released from the tissue and cell, binds to specific and saturable sites, initiates specific effector mechanisms and, finally, is inactivated (6). Of these criteria, most are strongly supported by the data. The least well-documented criterion is the release of the compound from the tissue and cell; additional work is needed in this area.

Side by side, a parallel group of researchers led by Paul J. Davis, were also exploring the nongenomic actions of THs in plasma membranes of other cell types, including RBC, immune cells, cancer cells, endothelial cells and in other systems including other species.

Hercbergs et al. reported that physiological levels of thyroxine (T4), and 3,3’,5’-triiodothyronine (reverse-T3), but not 3,5,3’-triodothyronine (T3), have nongenomic mechanisms of regulation of tumor cell proliferation, anti-apoptotic mechanisms, control over tumor-related angiogenesis and radio-resistance in just-diagnosed cancer patients. These nongenomic regulatory mechanisms are mediated via a plasma membrane thyrointegrin receptor, integrin αvβ3, which is typically expressed in proliferating cancer cells and vascular endothelium compared to normal nonmalignant cells.

Hercbergs et al. showed a similar nongenomic involvement of plasma membrane thyrointegrin receptor, integrin αvβ3, in breast cancer patients. They documented a T4-dependent proliferation of estrogen receptor (ER)-positive breast cancer cells. Occurrence of T4-dependent activation of thyrointegrin receptor during proliferation of triple-negative ER cells also suggests presence of more than one nongenomic mechanism of action for THs. Maintenance of euthyroidism is suggested to manage T4-induction of ER -positive breast cancer cells.

The relationship between genomic and nongenomic mechanisms was further investigated in the sea urchin *Strongylocentrotus purpuratus* by Taylor et al. In these studies, the first in an invertebrate organism, nongenomic mechanisms were measured by binding of fluorescently labeled T4 to membranes prepared from the sea urchins at various developmental stages by differential centrifugation. The gastrula stage showed the most robust binding. T4 showed the highest affinity for the receptor, but T3 also inhibited the binding of the labeled T4. rT3 was inactive, yet Arg-Gly-Asp (RGD), the integrin binding motif, displaced labeled T4. Genomic mechanisms were evaluated by RNA-seq experiments. In contrast to the case in mammalian brain, genomic mechanisms in the sea urchin occurred later in development than nongenomic mechanisms.

The articles in the current Research Topic emphasize the diversity and multiplicity of nongenomic mechanisms in various tissues and organisms. The research area initiated by Dratman is still not complete and considerable further investigation is required.

## Author contributions

PS: Conceptualization, Writing – original draft, Writing – review & editing. JM: Conceptualization, Writing – original draft, Writing – review & editing.
